# Short Communication: What interest and influence do Directors of Public Health in London have in local gambling policy?

**DOI:** 10.1016/j.puhip.2025.100635

**Published:** 2025-06-25

**Authors:** Jenny Blythe

**Affiliations:** NIHR Doctoral Fellow, London School of Hygiene and Tropical Medicine, UK

**Keywords:** Gambling harms, Licensing, Public health, Local government

## Abstract

**Objectives:**

Gambling harms are increasingly conceptualised as a public health issue, affecting not only the individual who gambles but those close to them and to wider society too. However, the Gambling Act 2005 gives public health teams no statutory role in decisions by local councils on ‘land-based’ gambling licensure, unlike alcohol premises licensing where public health teams are a responsible authority. We surveyed Directors of Public Health in London to gain understanding of their interest and influence in local gambling policy.

**Study design:**

a survey was created in conjunction with representatives from London public health teams and disseminated via an online platform. It was sent to Directors of Public Health in all 32 London boroughs.

**Methods:**

Survey questions were a mixture of fact-finding questions about the public health teams structure (including staff number) and Likert scale questions about their interest and influence in gambling policy, using comparator questions with alcohol policy (where public health teams have a formal role).

**Results:**

the response rate for the survey was 28 %. The place of public health teams within individual councils varies widely, as does the number of Full-Time Equivalent staff members. There was positive correlation between a public health teams perceived influence on alcohol and gambling policy. Public health teams identified a wide number of existing effective partnerships within their organisations.

**Conclusions:**

The findings suggest that it is public health team integration rather than pure legislative factors that influence their involvement in local gambling policy. Existing effective partnerships, particularly licensing and planning, should be harnessed to further integrate public health teams into decision-making.

## 1 Introduction

English councils act as licensing authorities for certain types of premises, with powers to approve, revoke, and set conditions for their operations. Many of these decisions have implications for public health, but the involvement of public health teams varies. Thus, these teams act as a “responsible authority” for alcohol licencing and must be informed of new applications [1], but the Gambling Act 2005 gives them no statutory role in decisions by councils on ‘land-based’ gambling [2]. This distinction is now being questioned as gambling harms are increasingly conceptualised as a public health issue, affecting not only the individual who gambles but those close to them and to wider society too [3].

We surveyed Directors of Public Health in London as part of a larger study of how local government might strengthen public health responses to gambling harms. We sought information and their perspectives on the place of public health teams within each local council (or “borough”) and the interest and influence local public health teams had on gambling policy locally.

## 2 Methods

The survey drew on findings from an earlier study that revealed how placement of public health teams within local government varied [4]. The initial questions was reviewed by five public health teams in boroughs across London, after which questions were added to elicit similarities and differences in the approaches taken by public health teams to gambling and alcohol, while recognising the statutory difference in responsibilities as described above. The final questionnaire comprised 20 questions.

The university's ethics committee approved the survey in January 2022 (Ref 26646). The instrument was hosted using an online survey platform and distributed as a hyperlink. All 32 Directors of Public Health in London were sent an invitation to complete the survey and data collection took place during March and April 2022.

## 3 Results

Nine of the 32 boroughs responded (response rate 28 %). Five (56 %) responses were from ‘inner’ London boroughs as defined by the Local Government Act 1963 [5]. The number of Full-Time Equivalent (FTE) staff within public health teams varied from 2 to 46, with a mean of 37 and median of 30. Four (44 %) reported that a public health team member had a formal position on their borough's licensing committee. There was considerable variation in placement of public health teams within authorities. Six of the nine (66 %) respondents were in public health teams that were part of another directorate, two of the nine (22 %) were their own distinct directorate and the remaining respondent's team was based outwith the borough.

Six of the nine (66 %) respondents identified gambling harms as a priority for their local authority and for the public health teams. Five had designated someone to lead on gambling in their public health team, three from inner London boroughs.

**Fig. 1:**
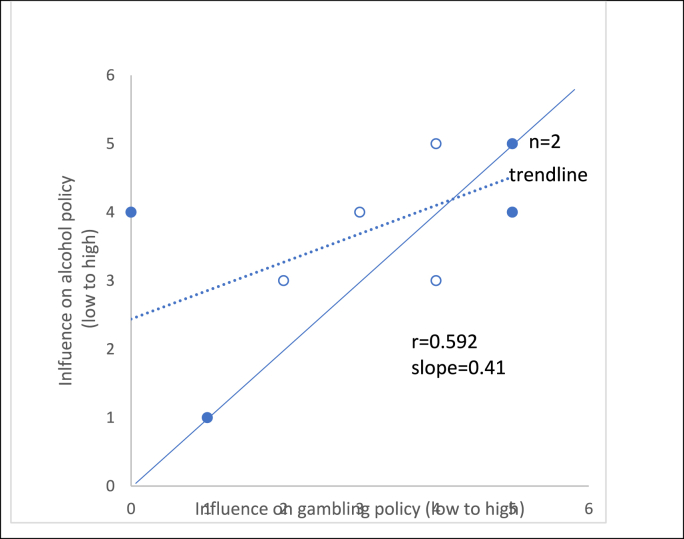
Perceived influence of public health teams on licensing of gambling and alcohol premises Of note: the correlation coefficient and slope are provided for completeness but should not be overinterpreted given small numbers. Open circles are outer London, closed circles are inner London.

The extent to which Directors of Public Health felt they could influence licensing decisions on gambling and alcohol varied considerably but was with a few exceptions was about the same for both, although a few felt they had slightly greater influence on alcohol licencing (Fig. 1).

Respondents did report having effective partnerships with other teams in their councils, most often mentioning planning and licensing teams (each cited by 4 respondents), but also social care, housing, the voluntary sector and academic institutions (all cited by 2 respondents each).

## 4 Discussion

This small study provides insights into local public health responses to gambling harms. Across the nine responding boroughs, the size and placement of public health teams varied considerably, as did whether gambling harms were seen as a priority for the council or its public health team. This was at a time when they are gaining more recognition as a public health issue, although change is slowed by how the industry and official policy has succeeded in framing gambling, incorrectly, as a largely harmless leisure activity, helped by the industry's domination of funding of research, education and treatment [6].

The wide range of staff employed in public health is striking and the smaller ones face obvious constraints in responding to health threats, including those caused by gambling.

Although inner London boroughs tend to be more deprived that outer ones, and there is a well-recognised link between gambling harms and deprivation [7,8], their public health teams were no more likely to have someone in their public health team allocated to gambling.

Most of those who reported gambling to be a priority for their local authorities and/or public health teams had allocated someone to it. However, caution is needed as those that had designated a gambling lead are likely to have been more willing to respond to the survey.

The positive correlation between public health teams’ perceived influence on gambling and alcohol licensing, even though they only have a statutory role in the latter, may say something about how embedded public health are within the licensing process, rather than with the specific product.

Finally, respondents reported a range of existing effective working relationships in local government relevant to gambling, likely reflecting where they were placed within their organisations.

The response rate is low despite strenuous efforts to elicit responses. The two-step process imposed by the ethics committee (with consent forms needing to be received before sending a hyperlink to the survey) could have reduced responses, as well as time constraints facing respondents during the pandemic.

The survey cannot say why Directors of Public Health differ in the extent to which they considered gambling harms as a public health issue, pointing to a need for further research.

Finally, the situation may have changed since the survey was undertaken, given this issue has risen on the national public health agenda, and the Gambling Act White Paper has been published [9,10].

The findings of this survey suggest that having a public health team member allocated to gambling, embedding public health teams within the alcohol licensing process, and leveraging existing effective relationships, specifically with licensing and planning, may all help to strengthen a public health approach to addressing gambling harms within local government.

## Ethics approval and consent to participate

London School of Hygiene and Tropical Medicine (LSHTM) ethics committee approved the study in Jan 2022 (Ref 26646).

## Funding

This report is independent research funded by the 10.13039/501100000272National Institute for Health and Care Research ARC North Thames. The views expressed in this publication are those of the author and not necessarily those of the National Institute for Health Research and Care or the Department of Health and Social Care.

## Declaration of competing interest

The author declares no conflicts of interest.

This report is independent research supported by the 10.13039/501100000272National Institute for Health and Care Research ARC North Thames. The views expressed in this publication are those of the author(s) and not necessarily those of the National Institute for Health and Care Research or the Department of Health and Social Care.
